# Nickel(II) uranium(IV) tris­ulfide

**DOI:** 10.1107/S1600536813033680

**Published:** 2013-12-21

**Authors:** Matthew D. Ward, James A. Ibers

**Affiliations:** a Department of Chemistry, Northwestern University, 2145 Sheridan Rd, Evanston, IL 60208-3113, USA

## Abstract

Crystals of NiUS_3_ were obtained from the reaction of the elements Ni, U, S, and of GeI_2_ in a CsCl flux at 1173 K. Nickel(II) uranium(IV) tris­ulfide, NiUS_3_, has ortho­rhom­bic (*Pnma*) symmetry and crystallizes in the GdFeO_3_ structure type. The compound has a perovskite *ABQ*_3_-like structure, with U occupying the inter­stitial sites of a NiS_6_ framework. The U atoms are coordinated by eight S atoms in a distorted bicapped trigonal–prismatic arrangement. The Ni atoms are coordinated by six S atoms in a slightly distorted octa­hedral arrangement. The asymmetric unit comprises one U site (site symmetry .*m*.), one Ni site (-1), and two S sites (1 and .*m*.).

## Related literature   

Uranium chalcogenides of the composition *ABQ*_3_ are known for Sc, V–Ni, Pd, Ru, Rh, and Ba (for a review, see: Narducci & Ibers, 1998 ▶). These compounds all have perovskite-type structures with *A* atoms occupying eight-coordinate inter­stitial sites within a *BQ*_6_ framework. There are two subclasses of the *ABQ*_3_ structure, *viz.* GdFeO_3_ (*Pnma)* (Marezio *et al.*, 1970 ▶) and FeUS_3_ (*Cmcm)* (Noël & Padiou, 1976 ▶). Single-crystal refinements have been carried out for BaUS_3_ (Brochu *et al.*, 1970 ▶), CrUS_3_ (Noël *et al.*, 1975 ▶), FeU*Q*_3_ (*Q* = S, Se) (Noël & Padiou, 1976 ▶; Jin *et al.*, 2010 ▶), ScUS_3_ (Julien *et al.*, 1978 ▶), RhUS_3_ (Daoudi & Noël, 1987 ▶), PdUSe_3_ (Daoudi & Noël, 1989 ▶), and MnUSe_3_ (Ijjaali *et al.*, 2004 ▶). The unit cell of NiUS_3_ was determined previously from powder diffracton experiments (Noël *et al.*, 1971 ▶). For standardization of structural data, see: Gelato & Parthé (1987 ▶).

## Experimental   

### 

#### Crystal data  

NiUS_3_
*M* *_r_* = 392.92
Orthorhombic, 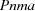

*a* = 6.8924 (3) Å
*b* = 8.7570 (4) Å
*c* = 6.0758 (2) Å
*V* = 366.72 (3) Å^3^
*Z* = 4
Mo *K*α radiation
μ = 50.68 mm^−1^
*T* = 100 K
0.09 × 0.09 × 0.08 mm

#### Data collection  

Bruker APEXII CCD diffractometer
Absorption correction: numerical (*SADABS*; Bruker, 2009 ▶) *T*_min_ = 0.093, *T*_max_ = 0.108
7334 measured reflections
748 independent reflections
728 reflections with *I* > 2σ(*I*)
*R*_int_ = 0.047

#### Refinement  

*R*[*F* ^2^ > 2σ(*F* ^2^)] = 0.018
*wR*(*F* ^2^) = 0.043
*S* = 1.36
748 reflections
29 parameters
Δρ_max_ = 2.80 e Å^−3^
Δρ_min_ = −1.15 e Å^−3^

### 

Data collection: *APEX2* (Bruker, 2009 ▶); cell refinement: *SAINT* (Bruker, 2009 ▶); data reduction: *SAINT*; program(s) used to solve structure: *SHELXS2013* (Sheldrick, 2013 ▶); program(s) used to refine structure: *SHELXL2013* (Sheldrick, 2013 ▶); molecular graphics: *CrystalMaker* (Palmer, 2009 ▶); software used to prepare material for publication: *SHELXL2013*.

## Supplementary Material

Crystal structure: contains datablock(s) I, New_Global_Publ_Block. DOI: 10.1107/S1600536813033680/wm2789sup1.cif

Structure factors: contains datablock(s) I. DOI: 10.1107/S1600536813033680/wm2789Isup2.hkl

Additional supporting information:  crystallographic information; 3D view; checkCIF report

## Figures and Tables

**Fig. 1.**
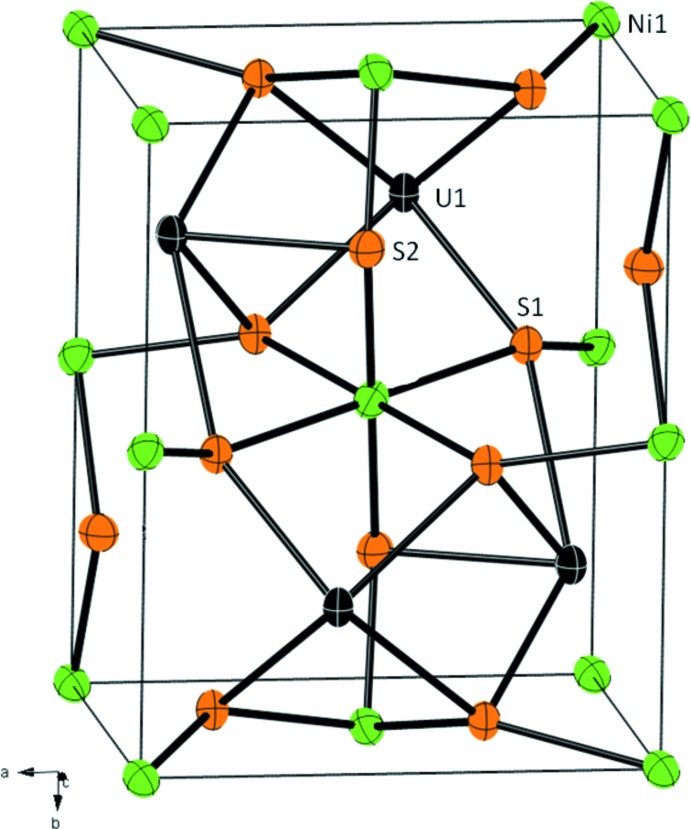
The unit cell of NiUS3. Displacement ellipsoids at the 95% probability level are shown.

**Fig. 2.**
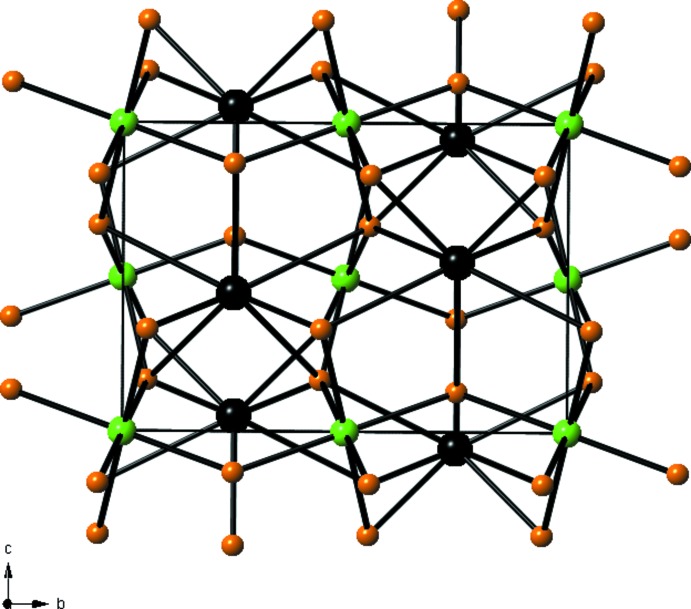
A packing diagram of NiUS3 viewed down the a axis. Ni atoms are green, U atoms are black, and S atoms are orange.

## supplementary crystallographic information

### 1. Comment

NiUS_3_ crystallizes in the orthorhombic space group *Pnma*. Its unit cell was previously determined (Noël *et al.*, 1971), revealing the compound to be isostructral with uranium compounds with analogous compositions. A number of uranium chalcogenides of the composition *ABQ*_3_ are known (for a review, see: Narducci & Ibers, 1998) and crystallize in two subclasses, *viz*. GdFeO_3_ (*Pnma*) (Marezio *et al.*, 1970) and FeUS_3_ (*Cmcm*) (Noël & Padiou, 1976). Most of the *ABQ*_3_ compounds crystallize in the three-dimesional GdFeO_3_ structure type. However, when *B* = Sc, Fe, or Mn, they crystallize in the layered FeUS_3_ structure type. Refinements based on single crystal data have been carried out for BaUS_3_ (Brochu *et al.*, 1970), CrUS_3_ (Noël *et al.*, 1975), FeU*Q*_3_ (*Q* = S, Se) (Noël & Padiou, 1976; Jin *et al.*, 2010), ScUS_3_ (Julien *et al.*, 1978), RhUS_3_ (Daoudi & Noël, 1987), PdUSe_3_ (Daoudi & Noël, 1989), and MnUSe_3_ (Ijjaali *et al.*, 2004).

The structure is composed of one U site, one Ni site, and two S sites. The uranium atoms are coordinated by eight S atoms in a distorted bicapped trigonal-prismatic arrangement. The Ni atoms are coordinated by six S atoms in a slightly distorted octahedral arrangement. The unit cell is shown in Figure 1 and a packing diagram is shown in Figure 2. There is no evidence of S—S bonding and thus formal oxidation states may be assigned as +II,+IV, and –II for Ni, U, and S, respectively. U—S distances range from 2.6666 (13) Å to 3.0088 (8) Å. These distances compare favorably with the U—S distances in the related compound RhUS_3_ (Daoudi & Noël, 1987). Ni—S distances range from 2.3386 (4) Å to 2.4739 (9) Å.

### 2. Experimental

NiUS_3_ was obtained from the reaction of U (0.126 mmol), GeI_2_ (0.063 mmol), Ni (0.126 mmol), and S (0.378 mmol) in a CsCl flux (0.445 mmol). The reactants were loaded into a carbon-coated fused-silica tube under an inert Ar atmosphere that was evacuated to 10 ^-4^ Torr. The tube was then flame sealed. It was placed in a computer-controlled furnace and heated to 1173 K in 12 h, held there for 6 h, cooled to 1073 K in 12 h and then held there for a further 96 h. The tube was next cooled at 5 K/h to 773 K and then to 298 K in 12 h. The reaction yielded black prisms of NiUS_3_ and black rectangular plates of NiU_8_S_17_ (Noël *et al.*, 1971). The crystals were washed with water and dried with acetone to remove excess flux. They are stable to both air and moisture.

### 3. Refinement

Atomic positions were standardized with the program *STRUCTURE TIDY* (Gelato & Parthé, 1987). The highest peak of 2.8 (3) e^-^/Å^3^ is 1.81 Å from atom S2 and the deepest hole of -1.2 (3) e^-^/Å^3^ is 0.96 Å from atom U1.

### Crystal data

**Table:**

NiUS_3_	*D*_x_ = 7.117 Mg m^−^^3^
*M**_r_* = 392.92	Mo *K*α radiation, λ = 0.71073 Å
Orthorhombic, *P**n**m**a*	Cell parameters from 3973 reflections
*a* = 6.8924 (3) Å	θ = 4.1–33.2°
*b* = 8.7570 (4) Å	µ = 50.68 mm^−^^1^
*c* = 6.0758 (2) Å	*T* = 100 K
*V* = 366.72 (3) Å^3^	Prism, black
*Z* = 4	0.09 × 0.09 × 0.08 mm
*F*(000) = 672	

### Data collection

**Table:**

Bruker APEXII CCD diffractometer	728 reflections with *I* > 2σ(*I*)
Radiation source: fine-focus sealed tube	*R*_int_ = 0.047
φ and ω scans	θ_max_ = 33.2°, θ_min_ = 4.1°
Absorption correction: numerical (*SADABS*; Bruker, 2009)	*h* = −10→10
*T*_min_ = 0.093, *T*_max_ = 0.108	*k* = −13→13
7334 measured reflections	*l* = −9→9
748 independent reflections	

### Refinement

**Table:**

Refinement on *F*^2^	0 restraints
Least-squares matrix: full	*w* = 1/[σ^2^(*F*_o_^2^) + (0.0149*F*_o_^2^)^2^]
*R*[*F*^2^ > 2σ(*F*^2^)] = 0.018	(Δ/σ)_max_ < 0.001
*wR*(*F*^2^) = 0.043	Δρ_max_ = 2.80 e Å^−^^3^
*S* = 1.36	Δρ_min_ = −1.15 e Å^−^^3^
748 reflections	Extinction correction: *SHELXL2013* (Sheldrick, 2013), Fc^*^=kFc[1+0.001xFc^2^λ^3^/sin(2θ)]^-1/4^
29 parameters	Extinction coefficient: 0.0039 (4)

### Special details

**Table:**

Geometry. All e.s.d.'s (except the e.s.d. in the dihedral angle between two l.s. planes) are estimated using the full covariance matrix. The cell e.s.d.'s are taken into account individually in the estimation of e.s.d.'s in distances, angles and torsion angles; correlations between e.s.d.'s in cell parameters are only used when they are defined by crystal symmetry. An approximate (isotropic) treatment of cell e.s.d.'s is used for estimating e.s.d.'s involving l.s. planes.

### Fractional atomic coordinates and isotropic or equivalent isotropic displacement parameters (Å^2^)

**Table:**

	*x*	*y*	*z*	*U*_iso_*/*U*_eq_	
U1	0.38141 (3)	0.2500	0.05064 (3)	0.00770 (8)	
Ni1	0.0000	0.0000	0.0000	0.00778 (13)	
S1	0.18039 (14)	0.05448 (9)	0.33217 (13)	0.00731 (15)	
S2	0.52930 (19)	0.2500	0.63121 (19)	0.0085 (2)	

### Atomic displacement parameters (Å^2^)

**Table:**

	*U*^11^	*U*^22^	*U*^33^	*U*^12^	*U*^13^	*U*^23^
U1	0.00525 (11)	0.00896 (10)	0.00890 (10)	0.000	0.00069 (5)	0.000
Ni1	0.0069 (3)	0.0081 (2)	0.0083 (2)	−0.0009 (2)	−0.0008 (2)	0.0002 (2)
S1	0.0057 (4)	0.0087 (3)	0.0075 (3)	0.0000 (3)	0.0000 (3)	0.0004 (3)
S2	0.0081 (6)	0.0079 (4)	0.0095 (5)	0.000	0.0023 (4)	0.000

### Geometric parameters (Å, º)

**Table:**

U1—S2^i^	2.6666 (13)	Ni1—S1^ix^	2.4180 (8)
U1—S2^ii^	2.7446 (12)	Ni1—S1	2.4180 (8)
U1—S1^iii^	2.7721 (9)	Ni1—S1^i^	2.4739 (9)
U1—S1^iv^	2.7721 (9)	Ni1—S1^vi^	2.4739 (9)
U1—S1^v^	2.7888 (8)	Ni1—U1^ix^	3.4349 (2)
U1—S1	2.7888 (8)	S1—Ni1^x^	2.4739 (9)
U1—S1^vi^	3.0088 (8)	S1—U1^i^	2.7722 (9)
U1—S1^vii^	3.0088 (8)	S1—U1^x^	3.0088 (8)
U1—Ni1	3.4349 (2)	S2—Ni1^x^	2.3386 (4)
U1—Ni1^viii^	3.4349 (2)	S2—Ni1^iii^	2.3386 (4)
Ni1—S2^i^	2.3386 (4)	S2—U1^iv^	2.6666 (13)
Ni1—S2^vi^	2.3386 (4)	S2—U1^xi^	2.7446 (12)
			
S2^i^—U1—S2^ii^	87.32 (2)	S2^i^—Ni1—S2^vi^	180.0
S2^i^—U1—S1^iii^	141.532 (18)	S2^i^—Ni1—S1^ix^	86.82 (3)
S2^ii^—U1—S1^iii^	87.85 (3)	S2^vi^—Ni1—S1^ix^	93.18 (3)
S2^i^—U1—S1^iv^	141.532 (18)	S2^i^—Ni1—S1	93.18 (3)
S2^ii^—U1—S1^iv^	87.85 (3)	S2^vi^—Ni1—S1	86.82 (3)
S1^iii^—U1—S1^iv^	76.29 (3)	S1^ix^—Ni1—S1	180.00 (4)
S2^i^—U1—S1^v^	78.58 (3)	S2^i^—Ni1—S1^i^	92.11 (4)
S2^ii^—U1—S1^v^	138.939 (19)	S2^vi^—Ni1—S1^i^	87.89 (4)
S1^iii^—U1—S1^v^	80.362 (19)	S1^ix^—Ni1—S1^i^	85.654 (14)
S1^iv^—U1—S1^v^	126.225 (15)	S1—Ni1—S1^i^	94.346 (14)
S2^i^—U1—S1	78.58 (3)	S2^i^—Ni1—S1^vi^	87.89 (4)
S2^ii^—U1—S1	138.939 (19)	S2^vi^—Ni1—S1^vi^	92.11 (4)
S1^iii^—U1—S1	126.225 (15)	S1^ix^—Ni1—S1^vi^	94.346 (14)
S1^iv^—U1—S1	80.362 (19)	S1—Ni1—S1^vi^	85.654 (14)
S1^v^—U1—S1	75.75 (3)	S1^i^—Ni1—S1^vi^	180.0
S2^i^—U1—S1^vi^	71.84 (2)	S2^i^—Ni1—U1^ix^	129.21 (3)
S2^ii^—U1—S1^vi^	69.082 (18)	S2^vi^—Ni1—U1^ix^	50.79 (3)
S1^iii^—U1—S1^vi^	139.987 (15)	S1^ix^—Ni1—U1^ix^	53.55 (2)
S1^iv^—U1—S1^vi^	70.81 (3)	S1—Ni1—U1^ix^	126.45 (2)
S1^v^—U1—S1^vi^	138.102 (15)	S1^i^—Ni1—U1^ix^	58.556 (19)
S1—U1—S1^vi^	69.890 (12)	S1^vi^—Ni1—U1^ix^	121.444 (19)
S2^i^—U1—S1^vii^	71.84 (2)	S2^i^—Ni1—U1	50.79 (3)
S2^ii^—U1—S1^vii^	69.082 (18)	S2^vi^—Ni1—U1	129.21 (3)
S1^iii^—U1—S1^vii^	70.81 (3)	S1^ix^—Ni1—U1	126.45 (2)
S1^iv^—U1—S1^vii^	139.987 (15)	S1—Ni1—U1	53.55 (2)
S1^v^—U1—S1^vii^	69.890 (12)	S1^i^—Ni1—U1	121.444 (19)
S1—U1—S1^vii^	138.102 (15)	S1^vi^—Ni1—U1	58.556 (19)
S1^vi^—U1—S1^vii^	124.80 (3)	U1^ix^—Ni1—U1	180.0
S2^i^—U1—Ni1	42.805 (10)	Ni1—S1—Ni1^x^	139.81 (4)
S2^ii^—U1—Ni1	101.60 (2)	Ni1—S1—U1^i^	87.36 (3)
S1^iii^—U1—Ni1	170.255 (18)	Ni1^x^—S1—U1^i^	132.47 (3)
S1^iv^—U1—Ni1	101.436 (18)	Ni1—S1—U1	82.22 (2)
S1^v^—U1—Ni1	93.784 (19)	Ni1^x^—S1—U1	85.92 (3)
S1—U1—Ni1	44.224 (18)	U1^i^—S1—U1	98.49 (2)
S1^vi^—U1—Ni1	44.546 (18)	Ni1—S1—U1^x^	96.93 (3)
S1^vii^—U1—Ni1	114.642 (18)	Ni1^x^—S1—U1^x^	76.90 (2)
S2^i^—U1—Ni1^viii^	42.805 (10)	U1^i^—S1—U1^x^	109.19 (3)
S2^ii^—U1—Ni1^viii^	101.60 (2)	U1—S1—U1^x^	152.25 (3)
S1^iii^—U1—Ni1^viii^	101.436 (18)	Ni1^x^—S2—Ni1^iii^	138.82 (6)
S1^iv^—U1—Ni1^viii^	170.255 (18)	Ni1^x^—S2—U1^iv^	86.41 (3)
S1^v^—U1—Ni1^viii^	44.224 (18)	Ni1^iii^—S2—U1^iv^	86.41 (3)
S1—U1—Ni1^viii^	93.784 (19)	Ni1^x^—S2—U1^xi^	106.53 (3)
S1^vi^—U1—Ni1^viii^	114.642 (18)	Ni1^iii^—S2—U1^xi^	106.53 (3)
S1^vii^—U1—Ni1^viii^	44.546 (18)	U1^iv^—S2—U1^xi^	136.28 (5)
Ni1—U1—Ni1^viii^	79.190 (5)		

Symmetry codes: (i) *x*−1/2, *y*, −*z*+1/2; (ii) *x*, *y*, *z*−1; (iii) *x*+1/2, −*y*+1/2, −*z*+1/2; (iv) *x*+1/2, *y*, −*z*+1/2; (v) *x*, −*y*+1/2, *z*; (vi) −*x*+1/2, −*y*, *z*−1/2; (vii) −*x*+1/2, *y*+1/2, *z*−1/2; (viii) −*x*, *y*+1/2, −*z*; (ix) −*x*, −*y*, −*z*; (x) −*x*+1/2, −*y*, *z*+1/2; (xi) *x*, *y*, *z*+1.
